# Hemolytic Crisis as the Initial Presentation of Hereditary Spherocytosis Induced by Parvovirus B19 and Herpes Virus Infection in a Patient with the Thalassemia Trait: A Case Report

**DOI:** 10.5505/tjh.2012.96977

**Published:** 2012-12-05

**Authors:** Meriç Kaymak Cihan, Hafize Gökçe, Meral Oruç, Lale Olcay

**Affiliations:** 1 Dr. Abdurrahman Yurtaslan Ankara Oncology Training and Research Hospital, Department of Pediatric Hematology, Ankara, Turkey; 2 Ankara University, School of Medicine, Department of Pediatric Hematology, Ankara, Turkey

**To the Editor, **

Human parvovirus B19 (PV-B19) causes erythema infectiosum, hydrops fetalis, and transient aplastic crisis in immunocompromised patients with chronic hemolytic anemia, arthralgia, and chronic pure red cell aplasia [1]. It may also cause autoimmune hemolytic anemia [2] (which presents as aplastic crisis with reticulocytopenia or increased erythropoiesis with reticulocytosis) [3], autoimmune thrombocytopenia/neutropenia, myelodysplastic syndrome, leukoerythroblastosis, hemophagocytic lymphohistiocytosis, and leukemia [1]. Transient aplastic crisis manifests as sudden exacerbation of anemia in patients with chronic hemolytic anemia, with severe reticulocytopenia lasting 7-10 days in the absence of erythroid precursors due to lysis of the precursors by PV-B19 [4]—the hallmark of which is giant pronormoblasts in the bone marrow [5]. 

Hemolytic crisis in hereditary spherocytosis (HS) is characterized by more pronounced jaundice than that in stable state of HS due to accelerated hemolysis and probable viral infection [6]. Herein we describe a patient that presented with hemolytic crisis as the initial manifestation of HS during PV-B19 infection—in contrast to the expectation of transient aplastic crisis [7]. This condition was attributed to coexistent herpes virus infection. The patient had a low mean corpuscular hemoglobin (MCH) and mean corpuscular hemoglobin concentration (MCHC) due to the thalassemia trait, which masked spherocytosis. 

A 15-year-old female was presented for evaluation of anemia. She had a 4-month history of progressive pallor, fatigue, scleral icterus, and dark urine. She has had intractable pallor since nearly 3 years of age Icterus was not noted until four months prior to her presentation to our hospital. Her brother also had pallor without icterus. Physical examination showed pallor, scleral icterus, and hepatosplenomegaly (spleen and liver extended the costal margins by 4 cm and 3 cm, respectively). 

Laboratory findings were as follows: hemoglobin level: 75 g/L; hematocrit: 24.1%; red blood cell count (RBC):4.11x10^12^/dL; platelet count: 279 x 10^9^/L; white blood cell count: 7.3 x 10^9^/L: mean corpuscular volume (MCV): 59.5 fL: mean corpuscular hemoglobin concentration MCHC 31.5 g/dl (N: 32.7-35.6 g/dl); mean corpuscular hemoglobin (MCH): 18.5 pg (normal range: 27.2-33.5 pg); red blood cell distribution width: 21% (normal range: 11.8%-14.3%); reticulocyte count: 2.2% (normal range: 0.6%-2.6%). Peripheral blood smear showed anisocytosis, poikilocytosis, hypochromia, and spherocytes. Direct and indirect Coombs tests were negative, and total/indirect bilirubin was 1.68/1.33 mg/dL, haptoglobin was <5.83 mg/dL (normal range: 36-195mg/dL), plasma hemoglobin was 7% (normal: <3), serum lactate dehydrogenase was 254 IU/L, and Hb A2 was 4.37%. Bone marrow aspiration showed erythroid hyperplasia, glucose-6-phosphate dehydrogenase, pyrimidine 5’ nucleotidase, pyruvate kinase, CD 55, and CD 59 evaluation of CD 55, and CD 59 on granulocytes by flow cytometry were normal. Collagen tissue disease markers were negative. Abdominal ultrasonography showed splenomegaly and elevated osmotic fragility. Herpes simplex virus HSV type 1-2 IgM was positive. PV-B19 PCR was 75.3 copies/mL in plasma, based on real-time PCR (normal: negative). 

The patient was diagnosed as hereditary spherocytosis and thalassemia trait, with coexistent PV-B19 and herpes virus infections.The hemoglobin level was found to have increased spontaneously to 88 g/L and her pallor and scleral icterus were found resolved at the end of in her evaluation made on 46^th^ day of admission At this time blood smear findings and mild indirect hyperbilirubinemia persisted and PV-B19 DNA showed 13 copies and HSV type 1-2 IgM was still positive. Viral serological tests may cross react with each other and we couldn’t perform HSV PCR. But HSV IgG was found to have increased from 6.15 to 113.2 RU/mL on the 46^th^ day of admission and this confirmed that PV-B19 and HSV infected the patient coexistently., 

The presented patient had mild HS, which manifested first as hemolytic crisis due to herpes virus infection, although infection by other viruses we didn’t test for could not be excluded. Her RBC count was within the lower limits of normal, which is unexpected in a patient with hemolytic anemia and the thalassemia trait. We think the normal RBC and reticulocyte counts, despite erythroid hyperplasia of the bone marrow, were indicative of repressed erythropoiesis due to PV-B19 infection. 

The presented case highlights the fact that, in the setting of chronic mild hemolytic anemia, patients in hemolytic crisis that have normal reticulocyte and RBC counts should be tested for PV-B19 infection and accompanying other viral infections . Clinicians should be aware that MCH and MCHC are not elevated in HS patients with thalassemia trait. 

**Conflict of Interest Statement**

None of the authors have any conflicts of interest, including specific financial interests, relationships, and/or affiliations, relevant to the subject matter or materials included.
